# Assembly Pathway of Hepatitis B Core Virus-like Particles from Genetically Fused Dimers*

**DOI:** 10.1074/jbc.M114.622035

**Published:** 2015-05-07

**Authors:** Kris Holmes, Dale A. Shepherd, Alison E. Ashcroft, Mike Whelan, David J. Rowlands, Nicola J. Stonehouse

**Affiliations:** From the ‡ School of Molecular and Cellular Biology, Faculty of Biological Sciences and Astbury Centre for Structural Molecular Biology, University of Leeds, Leeds LS2 9JT, United Kingdom and; § iQur Ltd, London Bioscience Innovation Centre, 2 Royal College Street, London NW1 0NH, United Kingdom

**Keywords:** hepatitis virus, mass spectrometry (MS), protein assembly, protein self-assembly, virus assembly, capsid, fused dimer, tandem core

## Abstract

Macromolecular complexes are responsible for many key biological processes. However, in most cases details of the assembly/disassembly of such complexes are unknown at the molecular level, as the low abundance and transient nature of assembly intermediates make analysis challenging. The assembly of virus capsids is an example of such a process. The hepatitis B virus capsid (core) can be composed of either 90 or 120 dimers of coat protein. Previous studies have proposed a trimer of dimers as an important intermediate species in assembly, acting to nucleate further assembly by dimer addition. Using novel genetically-fused coat protein dimers, we have been able to trap higher-order assembly intermediates and to demonstrate for the first time that both dimeric and trimeric complexes are on pathway to virus-like particle (capsid) formation.

## Teaser

**Background:** We studied assembly of HBV capsids using a genetically-fused dimer.

**Results:** Assembly intermediates composed of two, three and five copies of a fused dimer have been isolated.

**Conclusion:** Capsid assembly could progress using dimeric, trimeric, and pentameric intermediates.

**Significance:** The low abundance and transient nature of assembly intermediates make analysis challenging, however, the stability of the intermediates described here has overcome this.

## Introduction

**FIGURE 1.**
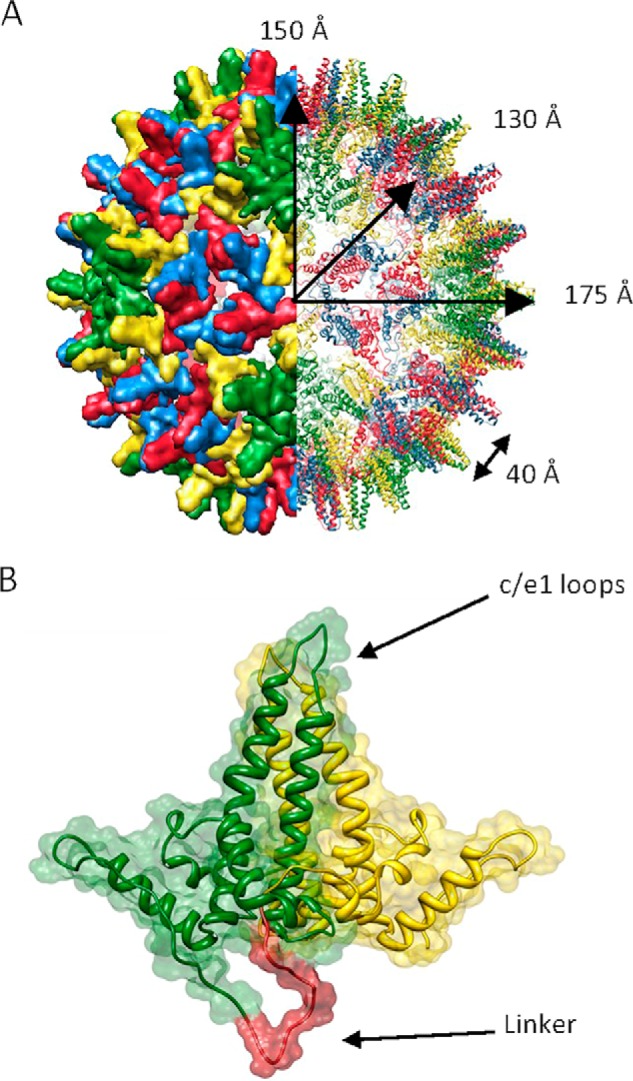
**HBV structure (PDB: 1QBT).** *A*, T = 4 capsid structure, space fill (*left*) and ribbon diagram (*right*). The capsid is composed of 4 quasi-equivalent subunits (shown in *red*, *yellow*, *blue*, and *green*) arranged as 120 dimers. It has an inner radius of 130 Å, is 20 Å thick and is punctuated by spikes that project 25 Å from the surface, giving a total radius of 175 Å. *B*, representation of the fused Cp, based on structure in *A*. The c/e1 loops are indicated and the two Cp monomers are shown in *green* and *yellow*, with the linker in *red*.

Viruses, such as hepatitis B virus (HBV),^2^ present as ideal candidates for the study of a range of biological processes, including macromolecular assembly. The core protein (Cp, also known as HBcAg) of HBV assembles, together with the pregenomic RNA (pgRNA) and the viral polymerase, into icosohedral nucleocapsids. Within these particles the pgRNA is reverse transcribed into DNA, the genomic form present in infectious virions (1). However, Cp alone is able to assemble into icosohedral virus-like particles (VLPs) *in vitro*, indistinguishable from the nucleocapsids formed in infected cells. These icosahedral capsids can be composed of either 90 or 120 dimers of Cp (*T* numbers 3 and 4, respectively). Cp is therefore in three possible quasi-equivalent environments in *T* = 3 capsids and four in *T* = 4 (Fig. 1*A*). The protein has two domains. Residues 1–149 form the assembly domain, necessary and sufficient for capsid assembly whereas the C-terminal nucleic acid binding domain is required *in vivo* (2). Evidence suggests that the latter domain is largely disordered (3), whereas the assembly domain is composed of five α-helices, lacking the canonical β-barrel structure prevalent in many capsid proteins. Its tertiary structure can be characterized as each monomer forming half of an upturned T (Fig. 1). Helices three and four comprise the dimer interface, or the T stalk. Helix five, together with the subsequent loop region, forms inter-dimer contacts that mediate capsid assembly at the 2- and 5-fold icosahedral axes. At the distal end of helix five, tyrosine-132 extends from the loop. This residue is essential for capsid assembly and contributes a significant proportion of buried hydrophobic surface at the inter-dimer interfaces (4, 5).

The quasi-equivalent environments are largely super-imposable with structural deviations being mostly confined to helix five, the proceeding loop and unstructured region (5). Assembly is not fully understood and although a wealth of kinetic and thermodynamic information exists (6–13), the pathway to capsid formation has yet to be elucidated in detail. Cp forms stable non-covalent dimers in solution and assembly can be initiated *in vitro* by increasing ionic strength (14). This is thought to act by inducing a conformational change in the dimer from assembly inactive to assembly active forms. This hypothesis is supported by the crystal structure of the Y132A assembly-defective mutant revealing subtle changes in structure compared with that in the context of the capsid (15). *In silico* assembly modeling suggests assembly progresses from a nucleation complex of a trimer of dimers (6). In addition, mass spectrometry analysis has demonstrated the presence of a number of species of up to 12 dimers, including a dimer of dimers and trimer of dimers (16). More recently, a combination of mass spectrometry and cryo-EM has revealed the presence of kinetically trapped incomplete capsids, which may represent intermediates later in assembly (13). While these observations are plausible (given that kinetic trapping is likely to trap assembly intermediates), assembly of the trapped species has not been followed on to the formation of intact capsids.

Previously, modified proteins have been employed to make investigation of macromolecular complexes more tractable. Pentamers of the HIV-1 capsid protein were isolated using artificially introduced disulfide linkages and, alternatively, template-driven assembly using fusion proteins (17). In addition, a C-terminal truncation of the E2 core of pyruvate dehydrogenase from *Bacillus stearothermophilus* was used to isolate and characterize a trimeric intermediate in the E2 cage assembly pathway (18). Indeed, the vast majority of previous studies of hepatitis B core assembly have used a truncated Cp incorporating the N-terminal assembly domain only. Removal of the C-terminal domain allows capsid disassembly/reassembly studies to be performed *in vitro*. We have incorporated a further modification into the assembly domain in order to investigate the capsid assembly process and to facilitate the use of these VLPs as generic scaffolds for antigen presentation (19). We have engineered a genetically-fused dimer (termed fused Cp) in which two copies of the Cp assembly domain (referred to as WT Cp) have been tethered with a C-N-terminal peptide linker. This is shown as a scheme in Fig. 1*B*. Both WT and fused Cp capsids have been produced in *Escherichia coli*.

Here, we have shown that disassembly reactions of capsids composed of dimers of WT Cp resulted in the release of non-covalent Cp dimers, as expected. In contrast, disassembly of the fused Cp capsids yielded several products, corresponding to a monomer, dimer and trimer of the fused Cp as well as possible higher-order species. Assembly reactions using these species successfully yielded fully assembled capsids. Furthermore, assembly reactions in which assembly of these species was allowed to progress in the presence of WT Cp dimers resulted in capsids that were a mixture of fused and WT Cp, consistent with the oligomeric complexes being incorporated into the newly formed capsids. These data suggest that the complexes are assembly-competent and therefore represent intermediates on pathway to fully assembled capsids.

## Experimental Procedures

### 

#### 

##### Plasmid Constructs

All constructs were cloned using the pET28b vector system. The fused Cp included a 5–7× GGS sequence, designed to link the C terminus of one assembly domain to the N terminus of the other (shown as a scheme for illustrative purposes in Fig. 1*B*). The construct (also termed tandem core CoHo) also included changes to the c/e1 loops to incorporate novel restriction sites. A full description of this construct and the generation of other fused Cp variants has been published elsewhere (19), together with a description of expression and characterization in both prokaryotic and eukaryotic systems and presentation of foreign epitopes.

##### Protein Expression and Purification

*E. coli* BL21 DE3 cells were grown to *A*_600_ 0.6–0.8 at 37 °C. Protein was expressed overnight at 16 °C following induction with 1 mm IPTG. Cells were lysed by French press and resulting lysates clarified by centrifugation at 50000 × *g* for 1 h. Protein was precipitated from clarified lysates by the addition of solid ammonium sulfate to 40%. The harvested precipitate was resuspended in 20 mm HEPES, pH 7.5, 250 mm NaCl, 5 mm DTT, and clarified at 10000 × *g* for 20 min followed by 20000 × *g* for 10 min. Protein was subjected to sedimentation through linear 20–60% sucrose density gradients in a Beckman AH629 rotor for 3 h at 4 °C followed by fractionation into 1 ml fractions. Fractions containing Cp, as demonstrated by SDS-PAGE, were dialyzed against 100 mm sodium bicarbonate, pH 9.6 and DTT (2 and 5 mm for monomeric and fused dimer samples, respectively). Dialysis resulted in ∼2.5-fold dilution of the protein. Solid urea was added to the dilute protein at 4 °C for 3 h to a final concentration of 3 or 4 m (for samples of WT or fused Cp, respectively) and subsequently concentrated to ∼2 AU at λ = 280 nm. The concentrated protein was briefly sonicated (2 × 5 s at 10 microns) then filtered through 0.22 μm membrane. The resulting protein was separated by size exclusion chromatography using a 26/60 Superdex-200 column equilibrated with 100 mm sodium bicarbonate, pH 9.7, and 5 mm DTT (50 mm sodium bicarbonate, pH 9.6 and 2 mm DTT for monomeric Cp). Protein elution was monitored at λ = 280 nm and peaks were analyzed by native and SDS-PAGE and by Western blotting and the fractions pooled accordingly.

##### Transmission Electron Microscopy

Negative stain transmission electron microscopy (TEM) was performed as described in Ref. 20 using 4% uranyl acetate. Samples were examined using a CM10 transmission electron microscope (Phillips, Guildford, UK).

##### Molecular Weight Determination using Size Exclusion Chromatography

Protein pooled from the original chromatographic separation was loaded onto a 26/60 Superdex-200 column and eluted at 1.0 ml min^−1^. The column had been calibrated using commercial molecular weight protein standards (Bio-Rad). These proteins were used to construct a standard curve, from which the oligomers masses were derived.

##### Non-covalent Mass Spectrometry

Spectra were acquired using a Synapt HDMS orthogonal acceleration quadrupole-time-of-flight mass spectrometer (Micromass UK Ltd, Waters Corp., Wilmslow, UK). Oligomer samples isolated from size-exclusion chromatography were button-dialyzed against 100 mm ammonium acetate, pH 6.8, whereas the fused Cp samples were analyzed in the same solution at pH 9.5. For the disassembly reaction analyzed without prior size-exclusion separation, fused Cp capsids were dialyzed into 4 m urea, 50 mm ammonium acetate, pH 9.5, 5 mm DTT, and incubated at 4 °C for ∼16 h. The sample was then dialyzed into 50 mm ammonium acetate, pH 9.5, 5 mm DTT. The samples were electrosprayed from gold/platinum-plated borosilicate capillaries fabricated in-house using a P-97 micropipette puller (Sutter Instrument Company, Novato, CA) and a sputter coater (Polaron SC7620; Quorum Technologies Ltd, Kent, UK). The electrospray capillary voltage was set at 1.7 kV, and the sample cone voltage at 40–60 V. To improve the resolution of the oligomer analyte signals, the voltage of the Transfer region of the mass spectrometer was increased to 50 V. The instrument had a source pressure of 3 mbar and a Trap gas (argon) flow rate of 2 ml min^−1^. Data were processed using the MassLynx (v 4.1) suite of software programs supplied with the instrument.

##### Assembly Reactions

Protein isolated from size exclusion chromatography was concentrated to a minimum of 0.2 mg ml^−1^ and subsequently dialyzed into HEPES pH 7.5. Assembly was initiated by the addition of sodium chloride to a final concentration of 750 mm. Reactions were incubated on ice for minimum of two hours, to ensure complete assembly. Capsids were visualized by TEM.

##### Chased Assembly Reactions

Samples of fused Cp oligomers were separated by size exclusion chromatography and mixed at a 1:1 ratio with samples of similarly purified WT Cp. Assembly of protein capsids was initiated with the addition of sodium chloride to 750 mm. Resulting assemblies were applied to a 10/30 Superdex 75 size exclusion column and the *V_o_* peak (representing assembled capsid) collected and fractions pooled. Capsids were immunoprecipitated from solution using antibody MAB16988 and protein G-Sepharose beads. This antibody reacts with the wild-type c/e1 loops within the context of the capsid but has no reactivity against disassembled protein or the fused Cp. Protein was analyzed by Western blot using Ab 10E11 (Abcam, Cambridge), reactive with all Cp protein samples.

## Results

### 

#### 

##### Capsids Composed of Fused Cp Disassemble into Oligomeric Complexes

Capsids composed of two different Cp variants (WT and fused Cp) were used in this study. Both Cps lacked the C-terminal domain, as described previously, for ease of disassembly/reassembly (21). The fused Cp consists of two WT Cp monomers linked between the C and N termini with a 5–7× GGS linker sequence. Capsids composed of WT or fused Cp were purified and subjected to disassembly. 3 m urea was sufficient to disassemble capsids composed of WT Cp (8). However, it was necessary to increase urea concentration to at least 4 m to facilitate disassembly of capsids composed of fused Cp.

**FIGURE 2.**
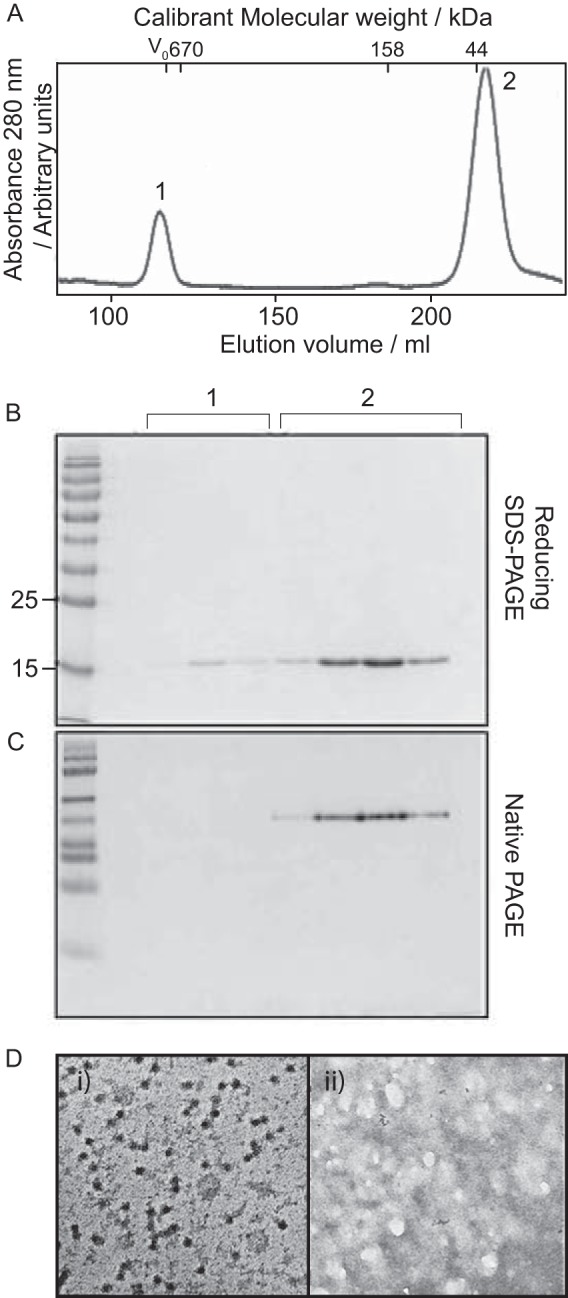
**Disassembly of capsids composed of WT Cp.** Protein at ∼2 mg/ml was disassembled with 3 m urea at pH 9.6. *A*, chromatogram of protein separation on a Superdex 200 column (capsid and Cp peaks are indicated as Peaks 1 and 2, respectively). *B*, reducing SDS-PAGE of peak fractions. Both peaks contained protein of the same molecular weight, corresponding to the expected size of WT Cp (17.3 kDa) by comparison to the standards shown (LHS). *C*, native PAGE of peak fractions. Protein from the capsid peak did not enter the gel matrix, however, the Cp peak is well resolved to a single species. *D*, TEM (negative stain) of (i) capsid peak (ii) Cp peak. Higher-order structures are visible in (i) however, (ii) is devoid of these. Bar, 100 nm.

Separation, using size exclusion chromatography (SEC), of WT samples resulted in two protein peaks, shown in the chromatogram, Fig. 2*A*. Both peaks contained Cp (predicted MW = 17.3 kDa) as shown by SDS-PAGE (Fig. 2*B*). However, protein from the first peak was unable to enter the matrix of a native-PAGE gel (Fig. 2) and was shown, by TEM, to contain particulate matter likely to be residual capsid not broken down by the disassembly process (Fig. 2*Di*). In contrast, material from the second peak was resolved as a single species by native PAGE (Fig. 2*C*). This sample contained no discernible higher-order structures by TEM (Fig. 2*Dii*).

**FIGURE 3.**
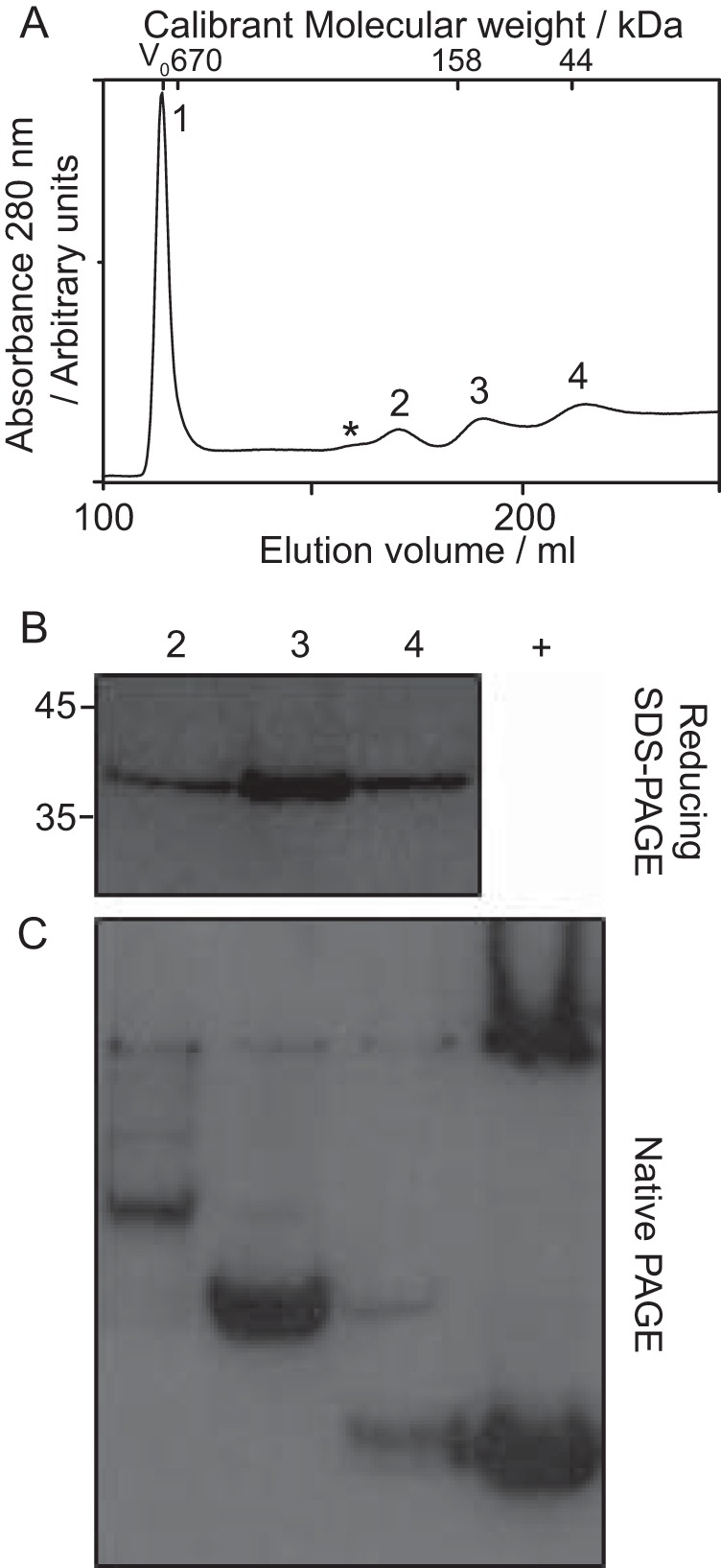
**Disassembly of capsids composed of fused Cp.** Capsids composed of fused dimers were disassembled with 4 m urea. *A*, size-exclusion chromatogram of fused Cp capsid disassembly reactions, resulting in a large peak consistent with non-dissociated capsid (Peak 1) and several other species labeled 2 to 4. *Asterisk* indicates additional low abundance species, which were not isolated. *B*, immunoblot of reducing SDS-PAGE of samples 2–4. All of the peaks contained protein of the same molecular weight, which was Cp antibody (10E11) reactive. Positions of standards are shown for reference. *C*, immunoblot of native PAGE of peak fractions. Proteins exhibited different migration patterns. The positive control (+) consists of Cp from disassembly of WT capsids.

In contrast, when the fused Cp capsids were subjected to the same process, at least four peaks resulted, as shown in Fig. 3*A*. The first (void volume) peak contained material corresponding to residual capsid but the ratio of disassembled material to residual capsid was reduced, indicating greater stability of capsids composed of fused Cp. Western blot analysis of SDS-PAGE of the peaks confirmed the presence of fused Cp in each, as expected (Fig. 3*B*). However, the peaks from the chromatogram migrated differently from each other when separated by native PAGE (Fig. 3*C*). Together, these results indicated that these species were oligomeric complexes of the fused Cp. We found no evidence of oligomeric complexes during disassembly of WT Cp capsids (Fig. 2). However, it remains quite possible that higher-order complexes were present (in equilibrium with the oligomers) at levels too low to detect.

Although the presence of the fused Cp oligomers could still be detected after a week, samples had a propensity to spontaneously aggregate and precipitate from solution, indicative that they may be in equilibrium with lower and higher-order oligomers. This was countered by maintaining the proteins in high pH buffer (a known assembly inhibitor (22)). Although dialysis into neutral pH buffer often resulted in precipitation; this was cleared by centrifugation prior to analysis of the protein.

##### Identification and Characterization of the Fused Cp Oligomeric Complexes

**FIGURE 4.**
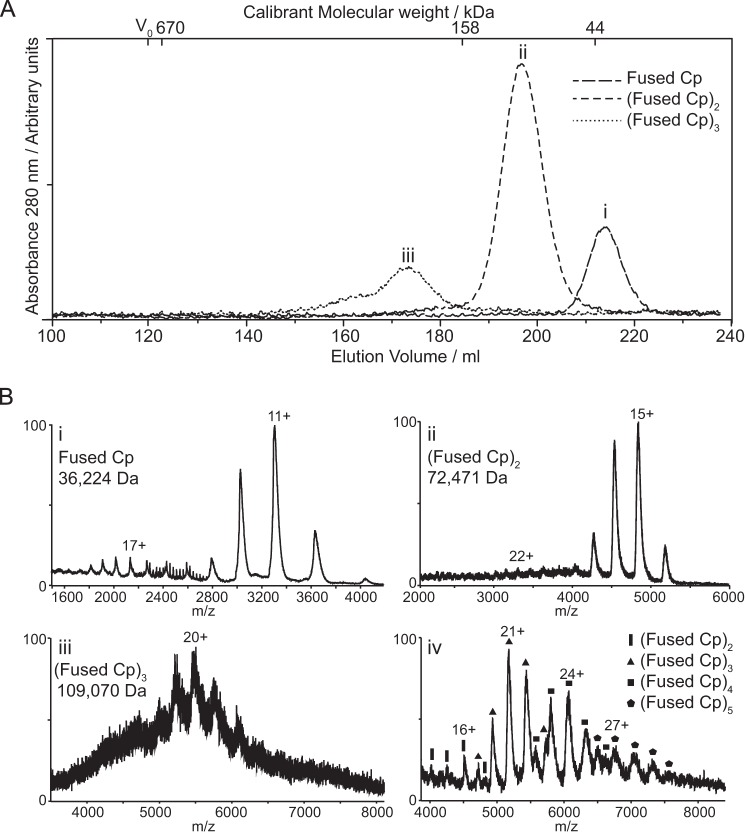
**Biophysical analysis of the oligomeric species.** *A*, characterization of the oligomers by size exclusion chromatography. Fractions of peaks 2–4 from the disassembly reaction (Fig. 3) were pooled and subjected to further SEC. Calibrated molecular weights were determined as 34.6 (i), 93.8 (ii), and 181.7 (iii) kDa. Labels i-iii correspond to those in part B. *B*, non-covalent ESI-MS of the oligomers. Derived masses are consistent with a monomer (i), dimer (ii) and trimer (iii) of the fused Cp. (iv). Non-covalent ESI-MS of a mixed sample of oligomers, showing species corresponding to up to five copies of fused Cp.

To determine the solution molecular weights of the fused oligomers, samples of each were separately re-analyzed using SEC on a calibrated Superdex-200 column, Fig. 4*A*. The elution profile was used to calculate the molecular masses of the oligomers based on the calibration curve of molecular weight standards. Extrapolated mass values were 34.6, 93.8, and 181.7 kDa. These equate to ∼1, 2–3, and 5 copies of the fused Cp (∼36 kDa). However, this technique can often lead to an over-estimation of mass. Therefore, these samples were also subjected to electrospray ionization-mass spectrometry (ESI-MS) under non-denaturing conditions (Fig. 4*B*) (23, 24). Using this technique, the fused Cp was clearly detected with a mass of 36,224 Da (Fig. 4*Bi*) and the oligomers observed were identified as dimers and trimers of the fused Cp, with measured masses of 72,471 and 109,070 Da, respectively (Fig. 4*B*, *ii* and *iii*). The high value obtained for the latter by SEC could be due to the presence of higher mass oligomers in the sample (Fig. 4*A*) but could also reflect differences in dynamics, as discussed previously (25). To characterize these low-abundance oligomers further, samples of fused Cp capsids were subjected to disassembly and ESI-MS analysis without SEC purification. In this mixed sample, oligomers with masses corresponding to two-five copies (inclusive) of the fused Cp were detected, with measured mass values of 72,400, 108,777, 145,384, and 182,935 Da, respectively (Fig. 4*Biv*). It is possible that the identification of the tetramer and pentamer of fused Cp in this sample was due to the absence of SEC purification, which can lead to the loss of material, resulting in lower concentration. Furthermore, the different abundances of the oligomers relative to those seen in the SEC chromatogram may be due to the different buffer conditions used. Nevertheless, the data agree qualitatively with SEC and provide a more accurate measurement of mass and therefore a more confident assignment of the oligomeric states of the fused Cp formed in the disassembly process.

##### Oligomers of Fused Cp Are on Pathway for Assembly into Capsids

**FIGURE 5.**
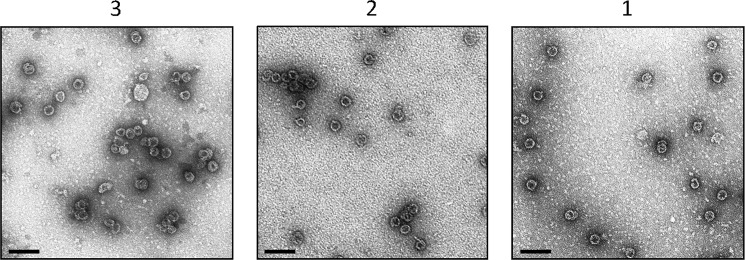
**TEM of oligomer assembly reactions.** Assembly was initiated by mixing samples of each complex with sodium chloride to a final concentration of 750 mm. Symbols 1, 2, and 3 relate to samples corresponding to fused Cp, and dimers or trimers of fused Cp, respectively. Micrographs (negative stain) of assembly reactions are shown, in the order corresponding to sample elution from size exclusion chromatography (see Fig. 3). The oligomers appear to be competent for assembly into capsids. Size bars, 100 nm.

Assembly reactions, akin to those previously documented (7), were carried out to determine if the samples corresponding to dimers or trimers of fused Cp were competent for assembly into capsids. Samples of each protein at ∼0.2 mg ml^−1^ were treated with NaCl to a final concentration of 750 mm and assembly allowed to proceed for minimum of 2 h on ice. Protein samples were then analyzed by TEM which showed the presence of capsids in each of the samples (Fig. 5). This suggested that the three oligomers were competent for capsid assembly and, furthermore, that the oligomers were on pathway and were therefore assembly intermediates. However, it is possible that contamination of samples with very small amounts of free fused dimer Cp could be responsible for the results observed.

**FIGURE 6.**
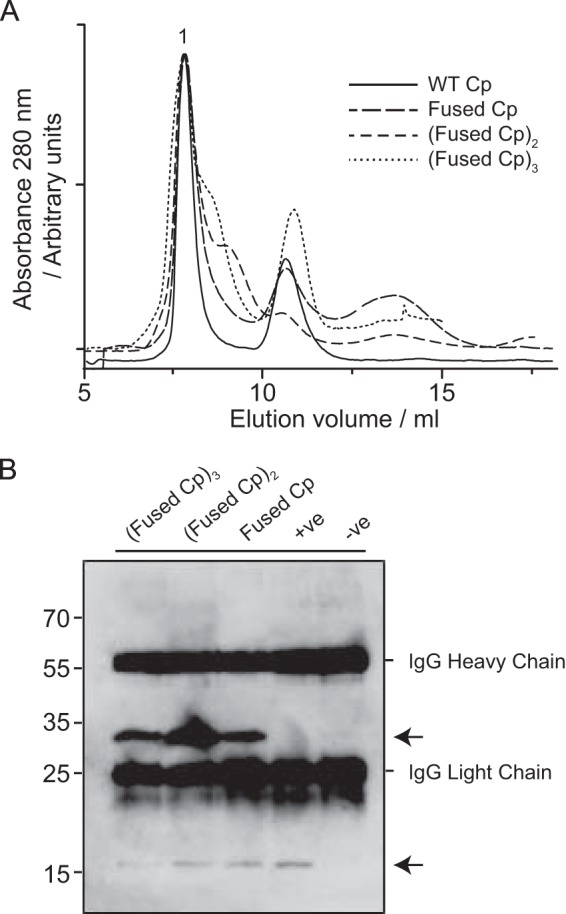
**Chasing the assembly of protein oligomers with WT Cp.** *A*, samples of fused Cp oligomers and WT Cp dimers were purified as above. Capsid peaks were discarded and samples representing fused Cp dimers and trimers mixed with WT Cp at 1:1 ratio. Assembly was initiated with the addition of sodium chloride to 750 mm. The resulting reactions were separated by size exclusion chromatography using a S75 column. *B*, void volume (capsid) peak (labeled 1) was analyzed. Capsids were immunoprecipitated from this using Mab 16988 (c/e1 loop specific and therefore unable to recognize fused Cp). Samples were analyzed by immunoblot (using the 10E11 antibody to recognize all Cp variants) showing these to be chimeric particles composed of both WT Cp and fused Cp. The positive control (+) is purified WT Cp from disassembly of WT capsids. (−) corresponds to a reassembly reaction with no Cp. *Arrows* indicate expected MW of WT Cp and fused Cp.

To confirm that the oligomers were indeed assembly-competent, we chased the assembly of each with WT Cp dimers. Samples of oligomers and WT Cp dimers were first purified using SEC, as described above. Capsid peaks were discarded and samples representing fused Cp, and dimers or trimers of fused Cp were mixed with WT Cp at 1:1 ratio (equimolar on the basis of mass). Reassembly was triggered with NaCl, as above and it was clearly demonstrated by SEC of the assembly reactions that all oligomers were assembly-competent (Fig. 6*A*). To characterize the composition of these capsids, we exploited the use of a monoclonal antibody to the c/e1 loop (MAB16988, Millipore). This antibody is able to recognize capsids composed of WT Cp (but not free Cp dimers) and is unable to recognize fused Cp under any conditions due to modifications of the c/e1 loops. We also employed an antibody (10E11) that was able to recognize all of these Cp proteins. Reassembled capsids (Fig. 6*A*) were isolated and immunoprecipitated with the c/e1 antibody, thereby removing any unassembled protein and ensuring that only capsids that incorporated the WT Cp would be isolated. To probe for the presence of the fused Cp in these reassembled capsids, samples were subjected to SDS-PAGE and immunoblot with the 10E11 antibody (Fig. 6*B*). A control sample of WT Cp dimers was included and was detected (after reassembly and immunoprecipitation) as a single Cp band, as expected. In contrast, the presence of both WT and fused Cp was detected in the capsids reassembled from the peak fraction samples, indicating clearly that both the dimeric and trimeric oligomers of fused Cp identified above were assembly-competent and constitute viable intermediate species on the pathway to capsid formation.

## Discussion

The assembly of macromolecular complexes is still poorly understood as pathway intermediates are often in very low abundance and transient in nature. Here, we have identified intermediates in the pathway of the assembly of the HBV capsid by exploiting novel assembly-competent fused dimers of the capsid protein together with a range of techniques including non-denaturing mass spectrometry. In previous studies using mass spectrometry to analyze WT Cp samples, the absolute masses of the capsids were determined to 0.1% tolerance and several oligomers were observed (16, 26). However, it was unclear whether these were on pathway to capsid assembly. Here, we have isolated and characterized similar oligomers. We have demonstrated previously that the fused Cp platform provides reduced flexibility (25) and may help stabilization of the intermediates, thus facilitating their detection and characterization. Consistent with this, Selzer *et al*. (12) also recently reported the importance of structural flexibility and that constraining the intradimer interface (by formation of disulfide bonds) could affect kinetics and thermodynamics of assembly. Interestingly, in addition to slower assembly, their data also demonstrated a decrease in stability of capsids formed from the more rigid WT Cp dimers. The unusual stability of the fused dimer oligomers here could be consistent with slower assembly kinetics, allowing their isolation and interrogation to a level not possible with other Cp variants. However, capsids composed of fused Cp appeared more stable relative to WT (at least in terms of urea denaturation).

We have reassembled capsids produced from samples of fused Cp oligomers (dimers or trimers) together with WT protein. These capsids were shown to contain a mixture of both WT Cp and fused Cp, and this result provides strong evidence that the oligomers represent complexes on pathway to capsid assembly. We have also shown the presence of species consistent with both tetramers and pentamers of Cp. Although these species may be assembly-competent, it has not been possible to undertake definitive experiments because of their low abundance.

**FIGURE 7.**
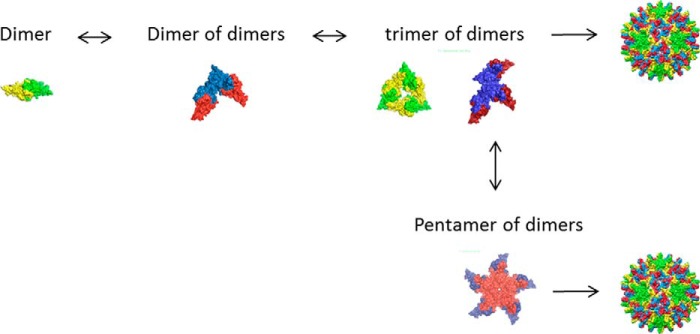
**Schematic of proposed assembly pathway, via either trimeric or pentameric intermediates.**

Previous studies have suggested a trimer of dimers as the nucleus of HBV capsid assembly (6, 16). It is also interesting to note that trimeric species have been identified as important assembly intermediates in other viruses (27). Indeed, from this work, it appears that this species is on pathway. However, assembly may also continue via tetrameric and pentameric intermediates. The structure of the trimer of dimers here remains unknown. It is possible that it is a closed ring of subunits (Fig. 7). However, it is tempting to speculate that it is an open structure, more competent to allow addition of further subunits *i.e.* corresponding to part of a 5-fold particle axis. The presence of putative tetrameric and pentameric intermediates (identified here by mass spectrometry) would strongly support this.

Using a related strategy, we previously demonstrated the presence of on-pathway intermediates in the assembly of bacteriophage MS2 capsids (28, 29). As here, it remained a possibility that capsid assembly occurs due to the disassembly of higher-order oligomers into dimers, which are then assembly-competent, rather than by the direct involvement of the oligomers. It should be noted that this study and the work presented here were carried out *in vitro* and in the absence of HBV pgRNA and the viral polymerase. It is therefore possible that different assembly pathway/s may occur *in vivo*, however, detailed studies of this nature are yet to be completed for any virus.

Our studies are continuing with the use of the HBV fused Cp system to present foreign antigens (via the modified c/e1 loops) as a novel vaccine platform, which we have termed tandem core.

## Footnotes

2 The abbreviations used are:

HBV: hepatitis B virus
Cp: core protein
VLP: virus-like particle
TEM: transmission electron microscopy.
